# Management of disseminated intravascular coagulation after thoracic endovascular aortic repair of type B aortic dissection: a case report

**DOI:** 10.1186/s12872-022-02768-6

**Published:** 2022-07-18

**Authors:** Hanbo Liu, Yi Liu, Jifu Lai

**Affiliations:** 1 Cancer Center, Department of Interventional Medicine, Zhejiang Provincial People’s Hospital (Affiliated People’s Hospital, Hangzhou Medical College), Hangzhou, Zhejiang China; 2 General Surgery, Cancer Center, Department of Vascular Surgery, Zhejiang Provincial People’s Hospital (Affiliated People’s Hospital, Hangzhou Medical College), 158 Shangtang Road, Hangzhou, 310014 Zhejiang China

**Keywords:** Aortic dissection, Disseminated intravascular coagulation, Thoracic endovascular aortic repair, Case report

## Abstract

**Background:**

Disseminated intravascular coagulation (DIC) is a critical and rare complication after thoracic endovascular aortic repair (TEVAR) of type B aortic dissection. The optimal treatment of aortic dissection-related DIC remains controversial.

**Case presentation:**

We herein describe the successful management of a 65-year-old man who presented with gingival bleeding and multiple subcutaneous petechiae and was proven to have DIC after TEVAR of aortic dissection. The patient had initially been discharged with improved laboratory tests after anticoagulation treatment followed by oral rivaroxaban for maintenance. However, he was readmitted with recurrent gingival bleeding 17 days later. The DIC was successfully controlled with a combination of anticoagulation and antifibrinolytics. After the patient was discharged, his treatment was switched to oral tranexamic acid and warfarin for maintenance. During a 15-month follow-up, the patient had no recurrence of hemorrhage symptoms and maintained stable coagulative and fibrinolytic parameters.

**Conclusions:**

Aortic dissection-related DIC requires long-term management under conservative treatment. The combination of warfarin and tranexamic acid may be a feasible method for long-term maintenance therapy.

## Background

Thoracic endovascular aortic repair (TEVAR) is regarded as the main therapy for acute type B aortic dissection. TEVAR is performed to cover the primary entry to the false lumen, which can decrease the pressure of the false lumen and enhance false lumen thrombosis [1]. Nevertheless, disseminated intravascular coagulation (DIC), a rare and potentially fatal complication of endovascular treatment, can develop because of the consumption of coagulation factors in aortic remodeling [2, 3]. Although several reports have described the treatment of DIC associated with aortic dissection, including surgery and medical therapies [4–7], the optimal treatment of aortic dissection-related DIC has not been established.

In this report, we describe a patient who developed DIC with hyperfibrinolysis after TEVAR of type B aortic dissection and was treated by a combination of anticoagulation and antifibrinolytics. Although treatment with warfarin in patients with aortic dissection-related DIC remains controversial, a therapeutic effect of the combined use of warfarin and tranexamic acid was observed in the present case.

## Case presentation

**Fig. 1:**
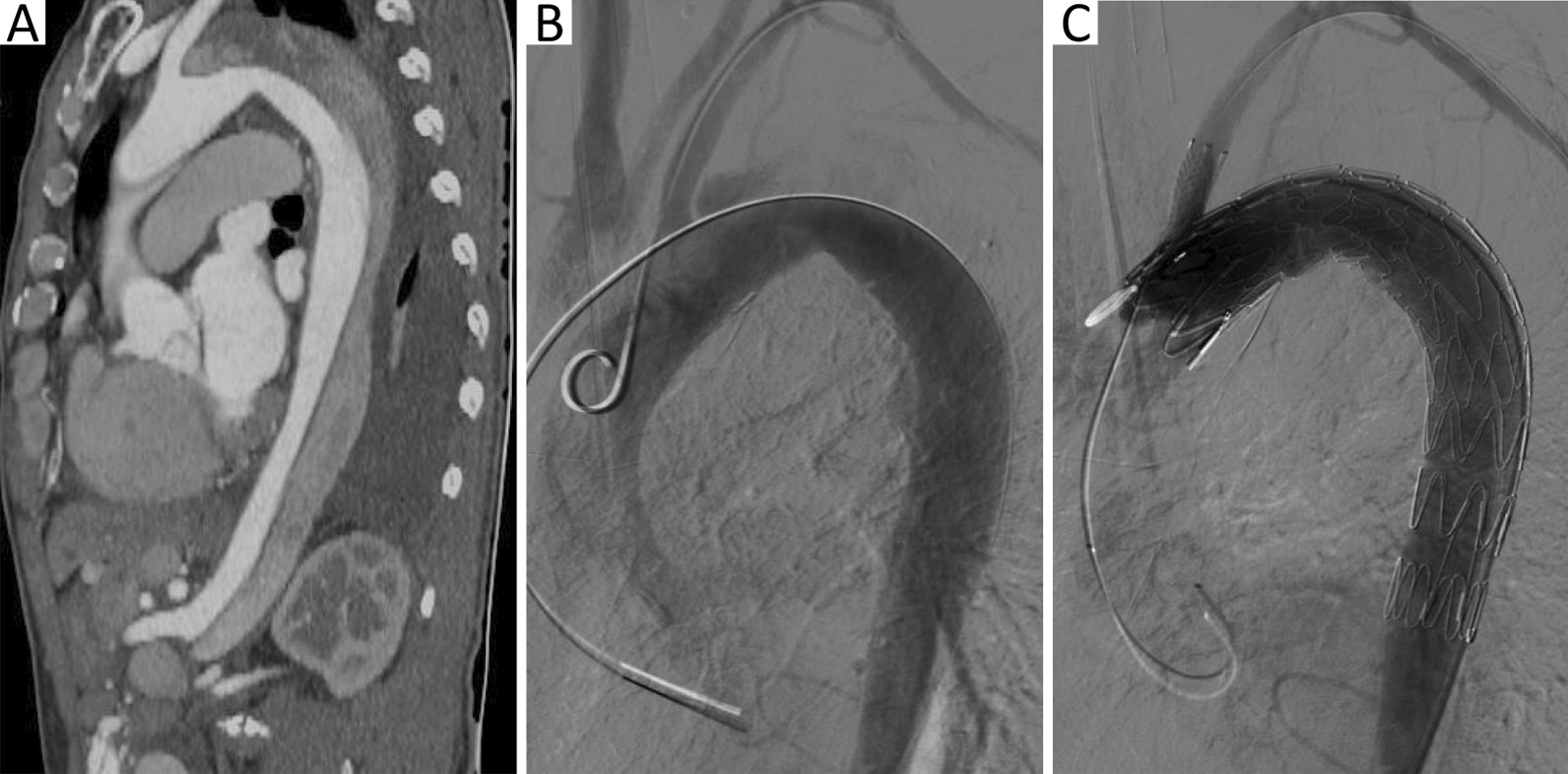
Historical images before admission. **A** CTA shows type B aortic dissection extending from the left subclavian artery to the bilateral iliac artery with partial thrombosis. **B**, **C** Images of angiography during TEVAR

**Fig. 2:**
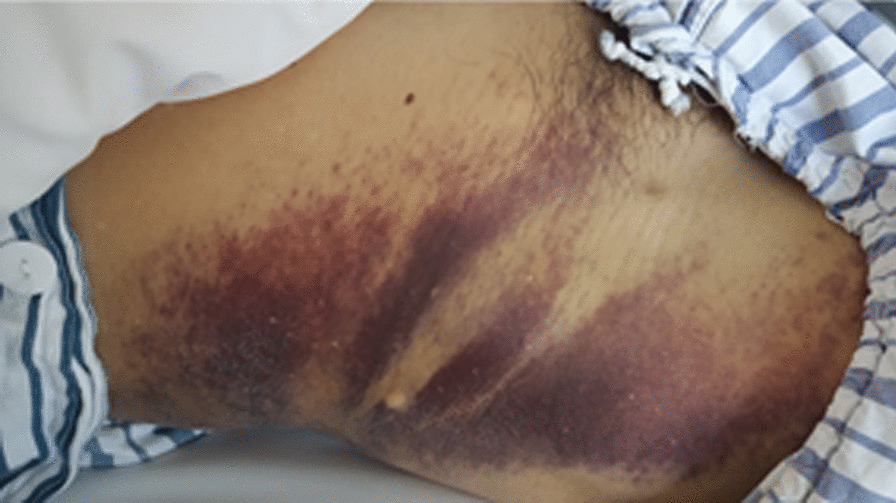
Physical examination shows multiple subcutaneous petechiae on the trunk and extremities upon admission

**Fig. 3:**
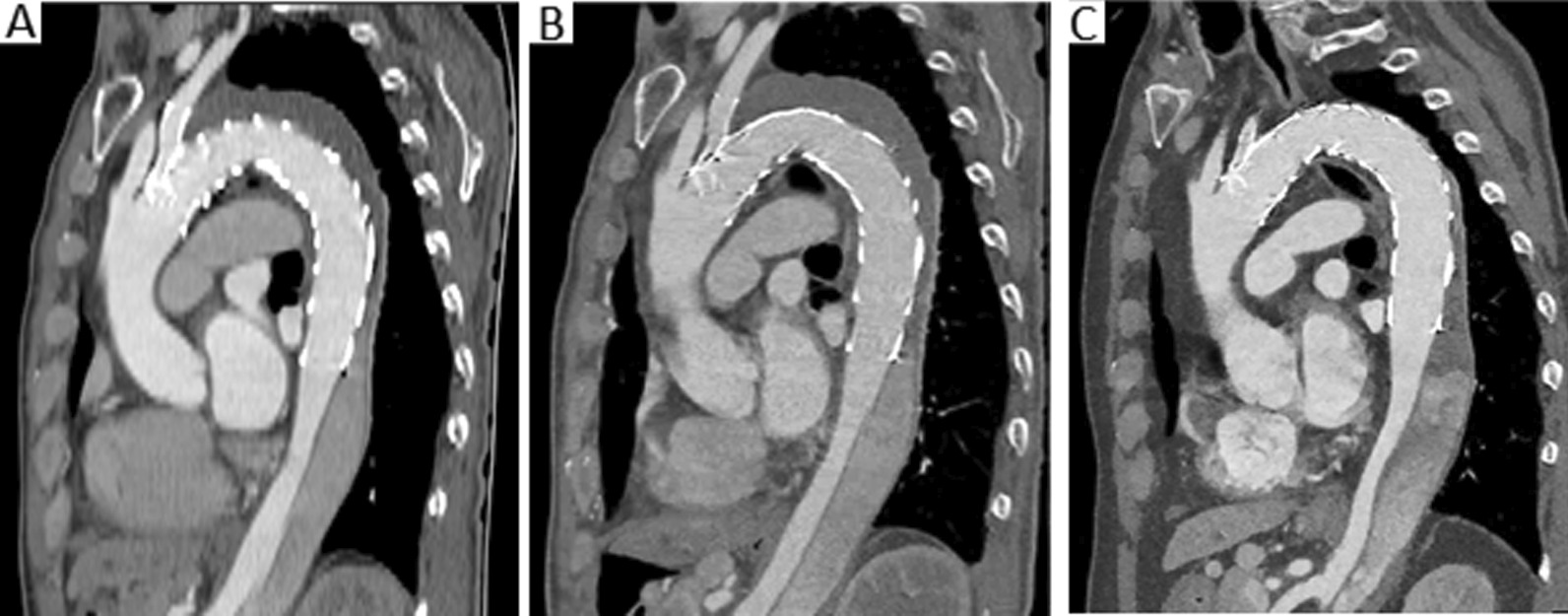
CTA on admission and during follow-up. **A** CTA shows persistent flow in the residual false lumen with partial thrombosis at the first admission. **B** CTA at readmission shows subtle progression of the thrombosis in the false lumen compared with the last admission. **C** CTA shows progression of the thrombosis in the false lumen without expansion at the 9-month follow-up

A 65-year-old man who had undergone branched TEVAR (Fig. 1) for type B aortic dissection 1.5 months previously was admitted to our hospital because of a 5-day history of gingival bleeding and multiple subcutaneous petechiae on his trunk and extremities (Fig. 2). His initial laboratory examination showed a hemoglobin concentration of 10.2 g/dL (reference range, 13–17.5 g/dL), platelet count of 37 × 10^9^/L (reference range, 125–350 × 10^9^/L), fibrinogen concentration of 50 mg/dL (reference range, 200–400 mg/dL), D-dimer concentration of 79.6 μg/mL (reference range, 0–0.55 μg/mL), activated partial thromboplastin time (APTT) of 45.9 s (reference range, 24.9–37.3 s), prothrombin time (PT) of 29 s (reference range, 9.8–13.2 s), and international normalized ratio (INR) of 2.66 (reference range, 0.52–1.2). Computed tomography angiography (CTA) on admission revealed persistent flow in the residual false lumen with partial thrombosis (Fig. 3A). No hematologic disorder was observed on bone marrow biopsy. The score for diagnosis of overt DIC as proposed by the International Society on Thrombosis and Hemostasis (ISTH) was 8. On the basis of the post-TEVAR clinical presentation of a decreased platelet count and fibrinogen concentration and an increased D-dimer concentration, the patient was considered to have DIC secondary to consumption of coagulation factors after TEVAR.

**Fig. 4:**
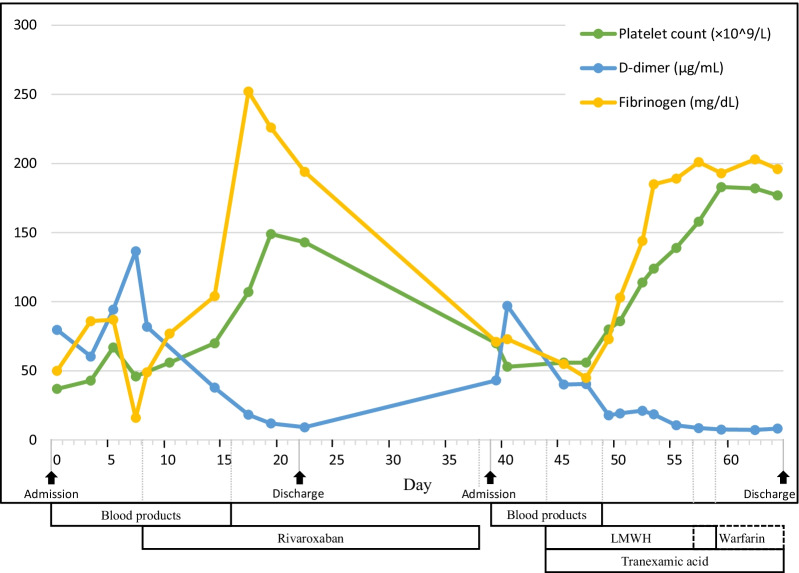
The clinical course of treatment for DIC. The timeline shows the response of DIC-related parameters to each intervention

To improve the patient’s coagulation function, we initially performed a transfusion of blood products including packed red blood cells (PRBC), fresh frozen plasma (FFP), and platelet concentrate (PC). However, the D-dimer concentration gradually increased and the fibrinogen concentration and platelet count remained low during the first week following supplementation with blood products (2 units of PRBC, 5 units of FFP, and 20 units of PC). Therefore, we decided to begin treatment with oral rivaroxaban, a direct oral factor Xa inhibitor, at a dose of 15 mg once a day. This strategy proved effective as shown by a gradual increase in the fibrinogen concentration and platelet count with a decrease in the D-dimer concentration (Fig. 4). Three weeks after admission, the patient was discharged with improved laboratory results (platelet count of 143 × 10^9^/L, fibrinogen concentration of 194 mg/dL, and D-dimer concentration of 9.24 μg/mL) and continued oral rivaroxaban at 15 mg/day for maintenance.

Unfortunately, 17 days after discharge, he was readmitted with recurrent gingival bleeding. Laboratory analysis showed a hemoglobin concentration of 10.6 g/dL (reference range, 13–17.5 g/dL), platelet count of 53 × 10^9^/L (reference range, 125–350 × 10^9^/L), fibrinogen concentration of 71 mg/dL (reference range, 200–400 mg/dL), D-dimer concentration of 43.14 μg/mL (reference range, 0–0.55 μg/mL), APTT of 48 s (reference range, 22.1–33.1 s), PT of 25.2 s (reference range, 9.8–13.6 s), and INR of 2.19 (reference range, 0.85–1.2). CTA on readmission showed subtle progression of the thrombosis in the false lumen compared with the last admission (Fig. 3B). The ISTH score of overt DIC was 7. Given the patient’s bleeding tendency, he was initially treated by supplementation with PRBC and FFP for 5 days. Nevertheless, his platelet count continuously decreased whereas his D-dimer concentration rapidly increased. Systemic treatment for DIC was then administered using subcutaneous low-molecular-weight heparin (LMWH), enoxaparin, at a dose of 50 mg twice a day and intravenous tranexamic acid at a dose of 2000 mg once a day. The patient’s laboratory analysis results rapidly improved thereafter (Fig. 4). The patient’s treatment was later switched to oral tranexamic acid (1000 mg/day) and warfarin (1 mg/day) for maintenance treatment. Finally, the patient was discharged with a normal platelet count (177 × 10^9^/L) and fibrinogen concentration (196 mg/dL), an observably decreased D-dimer concentration (8.28 μg/mL), and an INR of 1.29. CTA demonstrated progression of the thrombosis in the false lumen without expansion at the 9-month follow-up. During the 15-month follow-up period, the patient had no recurrence of hemorrhage symptoms and his laboratory indices remained relatively stable (platelet count, 138 × 10^9^/L; total bilirubin, 25.2 μmol/L; aspartate aminotransferase, 34 U/L; alanine aminotransferase, 30 U/L; fibrinogen, 124.2 mg/dL; D-dimer, 9.45 μg/mL; APTT, 28.1 s; PT, 21 s; INR, 1.53).

## Discussion and conclusions

DIC is an acquired syndrome characterized by intravascular activation of coagulation. It has various causes, but it is a relatively infrequent complication of aortic dissection [8, 9]. In general, most affected patients present with hemorrhage due to excessive fibrinolysis, but some may be asymptomatic because of compensatory mechanisms. The site of hemorrhage varies widely, ranging from gingival bleeding to multiple subcutaneous petechiae. Additionally, some cases of DIC are accompanied by hemorrhage of visceral organs [10]. The exact mechanism in this regard has not been elucidated. Most researchers consider that DIC after TEVAR of aortic dissection is secondary to consumptive coagulopathy caused by persistent retrograde flow in the false lumen [11, 12]. Identification and preventative substitution therapy for patients at high risk may help to prevent DIC.

No conventional therapy has been established for DIC with hyperfibrinolysis of aortic dissection. Particular attention should be paid to the underlying conditions to manage DIC [9]. Open surgery may be a radical treatment to correct the hemorrhagic diathesis but is quite invasive [13]. With rapid advances in endovascular technology, different methods for sealing re-entry tears have emerged [2, 4, 7, 12]. However, surgical or endovascular intervention may not be suitable for all patients, especially those with high operative risk. Under these circumstances, blood component therapy (e.g., FFP and PC) is recommended to supplement the coagulation factors and platelets that have been consumed [9]. Several medical strategies have shown effectiveness in controlling DIC due to aortic dissection, including anticoagulation [6, 14, 15], antifibrinolytics [8, 16, 17], and a combination of both [11, 18, 19]. Although anticoagulation is not usually recommended for patients with severe DIC-associated bleeding [9] because it may cause bleeding complications elsewhere, some reports have shown the effectiveness and safety of anticoagulation treatment for DIC in patients with aortic dissection. It is reasonable to presume that antifibrinolytics can control DIC with hyperfibrinolysis. Antifibrinolytics can promote the formation of false lumen thrombosis and terminate the consumption of coagulation factors once the false lumen is completely thrombosed [9]. Because of concern regarding obvious hemorrhagic diathesis, some researchers have treated DIC by combining blood components with antifibrinolytics (e.g., tranexamic acid and para-aminomethylbenzoic acid) and avoiding anticoagulation [8, 16, 17].

Direct oral anticoagulants (DOACs) such as rivaroxaban and apixaban have reportedly been used to successfully treat DIC [14, 20]. In the present case, although treatment with blood transfusion and oral rivaroxaban initially improved the patient’s DIC, the DIC recurred with maintenance treatment by anticoagulation alone (Fig. 4). We propose that anticoagulation treatment alone in patients with a persistently patent false lumen is insufficient because of the continuous consumption of coagulation factors and hyperfibrinolysis. Similarly, a previous report indicated that treatment with subcutaneous LMWH alone cannot completely restrain hypercoagulability and that such treatment may be dangerous if coagulation factors cannot be replenished [11]. A combination of anticoagulation and tranexamic acid has been shown to control DIC with hyperfibrinolysis [18]. In the present case, after we changed the strategy to a combination of anticoagulation and antifibrinolytics after supplementing coagulation factors, the recurrence of DIC was successfully controlled. Long-term management of DIC is necessary for patients with a persistently patent false lumen. After considering the burden of intravenous or subcutaneous administration and the cost of DOACs, we selected warfarin, a vitamin K antagonist, for maintenance therapy in this case. Although warfarin may be ineffective for remission of DIC secondary to aortic aneurysm [20], we found that the combination of warfarin and tranexamic acid was effective in long-term management.

In summary, management of aortic dissection-related DIC is necessary during long-term maintenance. Anticoagulation treatment alone may be insufficient if the patient has a persistently patent false lumen. A combination of warfarin and tranexamic acid may be a feasible method for long-term management.

## Data Availability

The datasets used and/or analysed during the current study are available from the corresponding author on reasonable request.

## Abbreviations

DIC: Disseminated intravascular coagulation
TEVAR: Thoracic endovascular aortic repair
APTT: Activated partial thromboplastin time
PT: Prothrombin time
CTA: Computed tomographic angiography
PRBC: Packed red blood cell
FFP: Fresh frozen plasma
PC: Platelet concentrate
LMWH: Low-molecular-weight heparin
DOACs: Direct oral anticoagulants
